# Histological grading evaluation of non-alcoholic fatty liver disease after bariatric surgery: a retrospective and longitudinal observational cohort study

**DOI:** 10.1038/s41598-020-65556-2

**Published:** 2020-05-22

**Authors:** Felipe David Mendonça Chaim, Lívia Bitencourt Pascoal, Fábio Henrique Mendonça Chaim, Bruna Biazon Palma, Tiago Andrade Damázio, Larissa Bastos Eloy da Costa, Rita Carvalho, Everton Cazzo, Martinho Antônio Gestic, Murillo Pimentel Utrini, Marciane Milanski, Elinton Adami Chaim, Raquel Franco Leal

**Affiliations:** 1 Department of Surgery, School of Medical Sciences, University of Campinas (UNICAMP), Campinas, Brazil; 2 IBD Research Laboratory, School of Medical Sciences, University of Campinas (UNICAMP), Campinas, Brazil; 3 Department of Pathology, School of Medical Sciences, University of Campinas (UNICAMP), Campinas, Brazil; 4 Laboratory of Metabolic Disorders, School of Applied Sciences, University of Campinas (UNICAMP), Limeira, Brazil

**Keywords:** Obesity, Chronic inflammation, Non-alcoholic fatty liver disease, Non-alcoholic steatohepatitis, Liver fibrosis, Obesity

## Abstract

Non-alcoholic fatty liver disease (NAFLD) is a chronic disease with several degrees of histological features which may progress to cirrhosis. Obesity is an important risk factor and although NAFLD has no specific pharmacological treatment, bariatric surgery has been associated with NAFLD regression in severely obese patients. However, few longitudinal histological studies support this finding. Therefore, firstly, a retrospective study was performed including clinical and histological data of 895 obese patients who underwent bariatric surgery. In addition, histological analyses of 30 patient’s liver biopsies were evaluated at two timepoints (T1 and T2). The retrospective analysis of the total number of patients revealed that the average body mass index (BMI) was 35.91 ± 2.81 kg/m^2^. The liver biopsies during bariatric surgery showed that 53.52% did not present NAFLD, 30.16% had NASH, 15.98% isolated steatosis and 0.34% liver cirrhosis. The median BMI of the longitudinal cohort decreased from 37.9 ± 2.21 kg/m^2^ at the time of bariatric surgery (T1) to 25.69 ± 3.79 kg/m^2^ after 21 ± 22 months after the procedure (T2). The prevalence of NAFLD in T1 was 50%, and 16.67% in T2. The histological area of collagen fiber was lower in T2 compared to T1 (p = 0.0152) in the majority of patients, which was also illustrated by immunohistochemistry for Kupffer cell and myofibroblast formation markers. These findings confirmed the NAFLD regression after bariatric surgery and, for the first time, showed the amelioration of these features using more accurate histopathological techniques.

## Introduction

Non-alcoholic fatty liver disease (NAFLD) is the most common chronic liver disease whose prevalence has been associated to the global obesity epidemic^1–3^. There are four clinical-pathological features which are usually followed by NAFLD course: non-alcoholic steatosis (NAFL), non-alcoholic steatohepatitis (NASH), NASH-related cirrhosis and hepatocellular carcinoma (HCC). Among them, obesity has been linked not only to initial stages of the disease, but also to its progression, leading to an increased morbidity and mortality. Moreover, NAFLD is strongly associated with insulin resistance, type 2 diabetes (T2D) and the incident cardiovascular disease (CVD)^4–6^. The worldwide prevalence of NAFLD and NASH in the general population has been estimated to span from 6.3–33% and 3–5%, respectively. This estimate is increasing with the rise in the incidence of obesity and T2D, so that the prevalence of the NAFLD may be over 85% among the morbid obese and 75.6% in patients with T2D regardless of obesity^1,5–7^. In the United States, the prevalence of obesity was 39.8% in 2016 and affected about 93.3 million of adults, while it has also been observed that the NAFLD/NASH is becoming the leading indication for liver transplantation^6,8^. So far, there have been no statistical data in the literature on the Brazilian obese population in relation to the prevalence and progression of the NAFLD.

Furthermore, no pharmacological agents have been approved for long-term treatment of NAFLD. The adoption of healthy lifestyles, such as dietary modifications, regular physical activity, and gradual weight loss, is considered the main clinical recommendation and an initial step for the management of NAFLD^1,2,5,6^. Bariatric surgery, by leading to significant weight loss and metabolic changes related to the release of incretins and adipokines, as well as decreasing chronic inflammation, can lead to a reduction of hepatic fat deposits. It is currently considered the gold standard treatment option for refractory morbid obesity, as it leads to a significant improvement and/or resolution of several obesity-related comorbidities^9–12^.

Therefore, this study aimed to characterize the NAFLD of obese Brazilian patients and to analyze the histologic evolution throughout the spectrum of NAFLD, while assessing the effects of bariatric surgery on the attenuation of this liver disease.

## Results

### Clinical and demographic characteristics of the bariatric surgery patients and evaluation of NAFLD – Retrospective cross-sectional analysis

**Figure 1:**
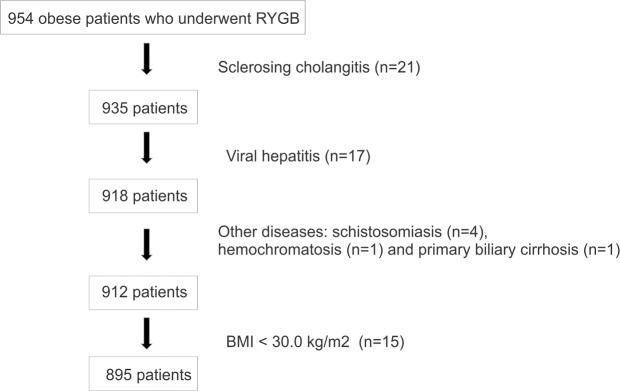
Patients enrolled in the observational study. Flowchart showing the patients who were excluded from the study. Obese patients operated by the Department of Surgery of the University of Campinas from 2008 to 2019. The exclusion criteria were based in age (<18 e > 80 years-old), BMI less than 30 kg/m^2^ at the time of surgery, lack of information in medical records and pre-existing liver diseases. RYGB: Roux-en-Y Gastric Bypass. BMI: body mass index.

This study included the clinical and demographic data of 895 Brazilian morbidly obese patients, of a total initial number of 954 patients who underwent RYGB bariatric surgery with liver biopsy during the procedure (Table 1). Twenty-one patients with sclerosing cholangitis, 17 viral hepatitis, 4 schistosomiasis, 1 hemochromatosis, 1 primary biliary cirrhosis and 15 patients with BMI under 30 were excluded from the analysis, as demonstrated in the Fig. 1. Eleven patients with benign tumors (adenoma, hamartoma, hemangioma and Von Meyenburg complex), 30 patients with surgical hepatitis and 166 patients with reactive liver in their anatomic pathology results were maintained in the analysis.

**Table 1:** Clinical and demographic characteristics of patients included in the retrospective study.

	**Prebariatric patients**
Number	895
Gender (M/F)	156/739
Age (years)	39.4 ± 10.2 (17.8-79.3)
BMI (kg/m^2^)	35.95 ± 2.81 (30.00-49.21)
Hypertension (yes/no)	387/508
Diabetes (yes/no)	163/732
ALB (g/dl)	4.5 ± 0.3 (2.2-5.4)
AST (U/L)	23.0 ± 13.6 (7-183)
ALT (U/L)	23.0 ± 28.8 (7-374)
AF (U/L)	65 ± 20.1 (11-227)
GGT (U/L)	19 ± 26.5 (4-438)
TB (mg/mL)	0.6 ± 0.3 (0.2-2.8)
PROT (g/dL)	7.2 ± 0.5 (5.0-9.2)
GLU (mg/dL)	83 ± 34.9 (47-485)
TGC (mg/dL)	92.50 ± 54.36 (32-559)
COL (mg/dL)	168.00 ± 35.31 (84-350)

Numerical variables are described as median [min. max] and categorical variables as absolute frequencies. M = male. F = female. BMI = body mass index. ALB = plasma albumin. AST = aspartate aminotransferase. ALT = alanine aminotransferase. AF = alkaline phosphatase. GGT = gamma glutamyl transferase. TB = total bilirubin. PROT = total protein. GLU = glucose. TGC = triglyceride. COL = total cholesterol.

The median age of the 895 patients was 38.1 ± 10.2 years-old and 739 (82.5%) were women. All patients included in the study had their BMI equal to or greater than 30 at the time of surgery, and the average BMI was 35.91 ± 2.81 kg/m^2^. The overall mortality rate was 0.33% (3 patients). Regarding the laboratory tests, plasma albumin (ALB), aspartate aminotransferase (AST), alanine aminotransferase (ALT), alkaline phosphatase (AF), gamma glutamyl transferase (GGT), glucose (GLU), total bilirubin (TB), total protein (PROT), triglyceride (TGC) and total cholesterol (COL) concentrations were analyzed (see Table 1). Of all patients we evaluated, 200 (22%) had normal serum levels of all blood tests, while 695 (78%) exhibited at least one altered serum levels when compared to the reference values of each test - as born out by 35% of the GLU, 21% of ALT, 16% of the COL, 13% of the GGT, 12% of the TGC, 10% of the TB, 9% of the PROT, 9% of the AST, 2% of the AF and 1% of the ALB blood tests. In addition, 163 patients (18% of all patients) were diagnosed with diabetes; and the serum glucose level of 66 patients (7,3% of all patients) was equal to or greater than 126 mg/dL at the time of bariatric surgery. Systemic arterial hypertension was verified in 387 patients (43% of all patients).

**Figure 2:**
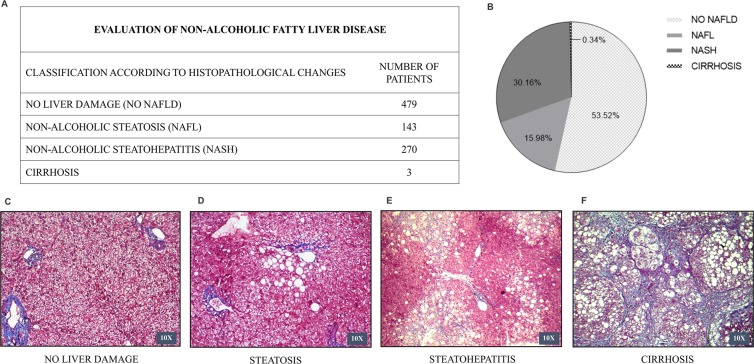
Histological evaluation of non-alcoholic fatty liver disease in the obese patients who underwent bariatric surgery – Retrospective cross-sectional analysis. Liver biopsies were performed during the bariatric surgery in the Clinical Hospital by the Surgery Department of the University of Campinasfrom 2008 to 2019. Number of patients included in the study = 895. (**A)** Classification of the NAFLD according to the histopathological changes. (**B)** Percentage of patients that were classified in each histological category: no liver damage, steatosis, steatohepatitis and cirrhosis. (**C–F)** Representative images of Masson’s Trichrome staining showing no histological change in the liver (**C**), hepatocytes displaying vacuoles related to triglyceride accumulation (macrogoticular steatosis) (**D**), hepatocellular ballooning (Mallory hyaline), macrogoticular steatosis and perisinusoidal fibrosis in acinar zone 3 (**E**), steatohepatitis with distortion of lobular architecture, collagen fibers highlighted in blue delineate hepatocytes forming septa that join portal structures and lobular center, in nodular transformation (cirrhosis) (**F**). 10× objective lenses were used as indicated in each image.

The anatomopathological findings for the histological diagnosis and fibrosis staging of NAFLD of liver biopsies were described as recommended by the American Association for the Study of Liver Diseases (AASLD) and the European Association for the Study of the Liver (EASL), which was based on an validated histological score: the FLIP (fatty liver inhibition of progression) Steatosis, Activity, and Fibrosis Score^13^, as shown in Fig. 2. Thus, of the entire evaluated group, 479 (53.52%) individuals had normal histological findings, 143 (15.98%) had NAFL; 270 (30.16%) had alterations compatible with NASH, such as the presence of steatosis, hepatocellular ballooning and lobular inflammation and 3 (0.34%) patients had cirrhosis. The histological score and the detailed results of the parameters used in this analysis are shown in the Supplementary Information Files.

### Clinical and demographic characteristics and evaluation of NAFLD after bariatric surgery – Retrospective longitudinal analysis

In a second step of this study, we compared clinical and histopathological findings of obese patients at two timepoints, at the time of bariatric surgery (T1) and after (T2) the procedure (n = 30). As shown in Table 2, 24 (80%) of the total patients were women. The median age at the time of the bariatric surgery (T1) was 41 ± 9 years-old and the median BMI was 37.90 ± 2.21 kg/m^2^. The median age at the time of the second surgery (T2) was 43 ± 9 years-old and the median BMI was 25.69 ± 3.79 kg/m^2^. Ten (33%) were ex-smokers. The median time between the liver biopsy collections during the surgeries (T1 and T2) was 21 ± 22 months, ranging from 3 to 82 months. There were no significant statistical differences regarding age, gender and smoking habit in the evaluated groups. However, there was a statistical difference between these groups regarding BMI (p < 0.001). There is no mortality after bariatric surgery in this subgroup of patients. However, there were mild to moderate complications in their follow-up such as incisional hernia (14 patients); gastric ring migration (6 patients); intestinal obstruction (abdominal adhesion in 2, and phytobezoar in another); anemia (3 patients), anastomotic ulcers (1 patient); depressive disorder (2 patients) and cholelithiasis (2 patients). One of these patients presented both incisional hernia and cholelithiasis.

**Table 2:** Clinical and demographic characteristics of patients at the time and after bariatric surgery and the control group.

	**Prebariatric patients** **(T1)**	**Postbariatric patients** **(T2)**	**P value**
Number	30	30	—
Gender (M/F)	6/24	6/24	0.9915
Age (years)	41 ± 9 (24-65)	43 ± 9 (25-67)	0.1136
Ex-smokers (yes/no/uninformed)	(10/18/2)	(10/18/2)	0.9921
BMI (kg/m^2^)	37.90 ± 2.21 (31.63-41.64)	25.69 ± 3.79 (19.95-34.50)	0.0000*
Percent total weight loss (%)	—	30 ± 11(11-52)	—
Hypertension (yes/no/uninformed)	(14/14/2)	(4/19/7)	0.0065*
Diabetes (yes/no/uninformed)	(7/21/2)	(1/22/7)	0.0592
Alcohol consumption (yes/no)	(0/30)	(0/30)	>0.999
ALB (g/dl)	4.3 ± 0.4 (3.4-5)	4 ± 0.6 (2.7-4.8)	0.0014*
AST (U/L)	21 ± 5.7 (12-36)	18 ± 8.2 (13-52)	0.0799
ALT (U/L)	21 ± 14.1 (10-71)	17 ± 10.1 (8-56)	0.0117*
AF (U/L)	63 ± 16.9 (36-105)	67 ± 22.6 (33-131)	0.7135
GGT (U/L)	20 ± 18.2 (9-70)	13 ± 14.8 (5-71)	0.0072*
AMYL (U/L)	37 ± 14.5 (21-79)	42 ± 19.6 (24-96)	0.3067
LIP(U/L)	27 ± 36.5 (5-199)	23 ± 23.6 (9-98)	0.5279
TB (mg/mL)	0.5 ± 0.5 (0.24-2.36)	0.5 ± 0.4 (0.18-1.96)	0.4137
PROT (g/dL)	7 ± 0.5 (5.7-8.1)	6.6 ± 0.7 (5.2-7.8)	0.0109*
GLU (mg/dL)	86 ± 21.1 (65-167)	83 ± 18.3 (73-148)	0.5752
TGC (mg/dL)	101.5 ± 42.8 (47-200)	69 ± 32.9 (35-186)	0.0216*
COL (mg/dL)	169.5 ± 28.5 (88-226)	145 ± 30.1 (52-187)	0.0031*
Time since last surgery (months)	—	21 ± 22 (3-82)	—

Numerical variables are described as median [min. max] and categorical variables as absolute frequencies. M = male. F = female. BMI = body mass index. ALB = plasma albumin. AST = aspartate aminotransferase. ALT = alanine aminotransferase. AF = alkaline phosphatase. GGT = gamma glutamyl transferase. AMYL = amylase. LIP = lipase. TB = total bilirubin. PROT = total protein. GLU = glucose. TGC = triglyceride. COL = total cholesterol. ^*^p < 0.05 T1 vs. T2. For “ex-smokers”, “hypertension” and “diabetes” variables: Pearson’s chi-squared test. For the other variables: Mann Whitney test and Dunn’s multiple comparisons test.

**Figure 3:**
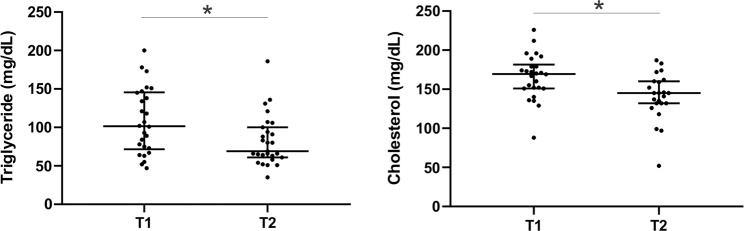
Triglycerides and cholesterol levels at the time of bariatric surgery (T1) and after (T2) the procedure. * p ≤ 0.05 T1 vs. T2. Mann Whitney test and Dunn’s multiple comparisons test.

All groups were evaluated for serum levels of liver function markers, which are expressed as median values and variations showed in Table 2 and represented in the Fig. 3. At the time of their bariatric surgery, 25 (83%) of the obese patients presented abnormal levels compared to the reference values - as born out by 37% of the GLU, 17% of the TGC, COL, TB and GGT tests, 13% of ALB and PROT results, 10% of ALT, 7% of lipase (LIP) and 3% of amylase (AMYL) blood tests. At the time of the second surgery (post-bariatric surgery), 27 patients (90%) presented a discrepancy, corresponding to 30% of the PROT, 20% of the GLU, 13% of the ALB, 7% of the AMYL and LIP and 3% of the TGC, TB, ALT, AST, AF and GGT blood tests. Significant statistical differences were found among the groups regarding ALT, PROT and TGC (p < 0.05), ALB, COL and GGT (p < 0.01), being decreased in T2 group compared to T1 (Table 2). Only 2 patients (6.6% of all 30 patients) and another 2 (6.6%) had serum glucose equal to or greater than 126 mg/dl at the time of (T1) and after (T2) bariatric surgery, respectively. The lipid levels at the two timepoints were also graphically represented in the Fig. 3.

**Figure 4:**
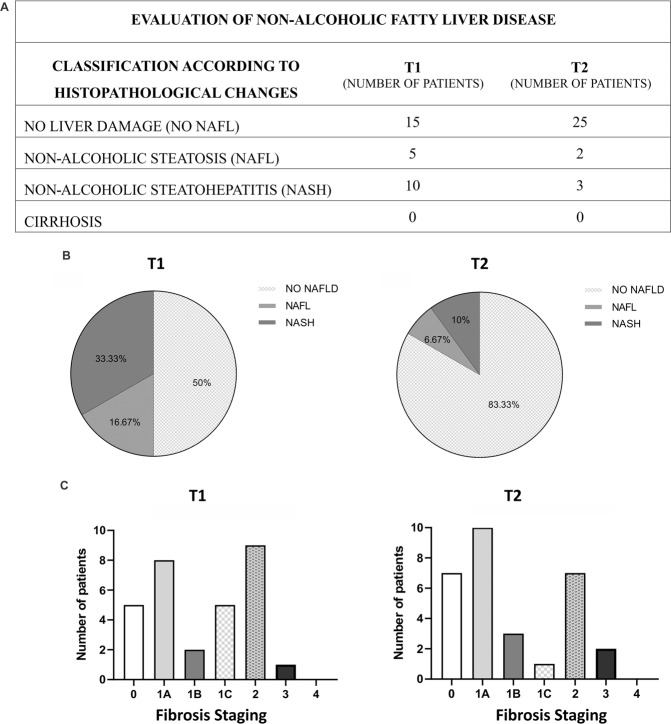
Evaluation of non-alcoholic fatty liver disease at the time of (T1) and after (T2) bariatric surgery. Liver biopsies were performed during the bariatric surgery and also during the second abdominal procedure which the patients were submitted in their follow-up (median of 22 months between surgeries). (**A)** Classification of the NAFLD according to histopathological changes of patients before (T1) and after (T2) bariatric surgery (n = 30). (**B)** Percentage of T1 and T2 patients that were classified in no liver damage (No NAFLD), non-alcoholic steatosis (NAFL), non-alcoholic steatohepatitis (NASH) and cirrhosis according to histopathological changes. (**C**) Fibrosis staging of T1 and T2 patients according to histopathological changes.

Liver biopsy evaluation was also performed, as shown in Fig. 4. The anatomopathological findings of the liver biopsies collected during bariatric surgery (T1) revealed 50% of NAFLD, with 33.33% NASH and 16.67% NAFL, while in T2, only 16.67% of patients presented NAFLD, being 10% NASH and 6.67% NAFL. The histological score and the detailed results of the parameters used in this analysis are shown in the Supplementary Information Files.

The stage of fibrosis was also determined by the anatomopathological findings based on the Fibrosis Score, recommended by the American Association for the Study of Liver Diseases (AASLD) and the European Association for the Study of the Liver (EASL)^13^. These findings were classified as: no fibrosis (0), zone 3 mild perisinusoidal (1 A), zone 3 moderate perisinusoidal (1B), portal or periportal fibrosis (1C), Zone 3 plus portal/periportal (2), bridging (3) and cirrhosis (4). At the time of bariatric surgery (T1), 5 patients (16.6%) did not present fibrosis, 8 patients (26.66%) had fibrosis at stage 1 A, 2 patients (6.66%) at stage 1B, 5 patients (16.66%) at stage 1C, 9 patients (30%) at stage 2 and 1 patient (3.33%) at stage 3. At timepoint T2, 7 patients (23.33%) did not present fibrosis, 10 patients (33.33%) had fibrosis at stage 1 A, 3 patients (10%) at stage 1B, 1 patient (3.33%) at stage 1C, 7 patients (23.33%) at stage 2 and 2 patients (6.66%) at stage 3.

### NAFLD regression after bariatric surgery: collagen fibers, myofibroblast formation and macrophage markers in the liver biopsies analysis

**Figure 5:**
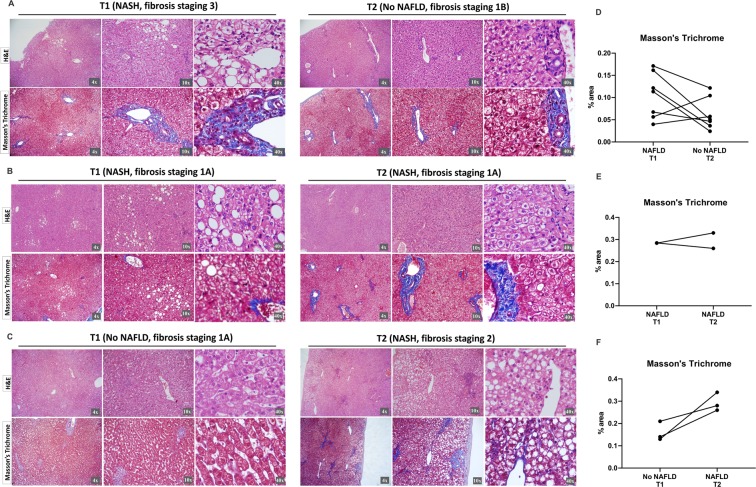
Histopathological analysis of liver biopsies at the time (T1) and after (T2) bariatric surgery showed NAFLD regression after the procedure. A-C. Representative images of hematoxylin and eosin (H&E) and Masson’s Trichrome staining of the T1 and T2 groups. Collagen fibers stained by the Masson’s Trichrome are shown in blue. **(A)** NAFLD regression (*p = 0.0152, n = 7). **(B)** NAFLD maintainance (n = 2). **(C)** Development of liver disease after bariatric surgery (n = 3). 4×, 10× and 40× objective lenses were used as indicated in each image. **(D–F)** Quantitative analysis of Masson’s Trichrome staining in T1 and T2 of patients who presented NAFLD in T1 and/or T2.

The hepatocytes with accumulated triglyceride vacuoles are the histological feature that ties together all of the various types of NAFLD^14^. We can readily observe this feature by hematoxylin and eosin (H&E) (10×) in the Fig. 5A (T1), B (T1 and T2) and C (T2), representing NAFLD regression, NAFLD maintainance and development of liver disease after bariatric surgery, respectively. Indeed, hepatocyte ballooning, the most characteristic feature of steatohepatitis (NASH)^14^, is shown by H&E (40×). These histological alterations were evidenced together in the majority of the morbidly obese patients with NAFLD who underwent bariatric surgery (T1), and in the minority of the patients in T2, as previously shown in the Fig. 4.

Moreover, representative photomicrographs of Masson’s Trichrome staining in T1 and T2 are shown in Fig. 5A–C. We can observe a significant decrease of collagen fibers in T2 compared to T1 (p = 0.0152, n = 7) (Fig. 5D), corroborating the NAFLD regression that occurred in the majority of obese patients after bariatric surgery in this paired cohort. Two patients presented NASH at T1 and showed no improvement at T2 (Fig. 5B,E). The interval between the surgeries of these 2 patients was 16 months, and one maintained a BMI > 30 after bariatric surgery. Finally, three patients who did not have NAFLD after bariatric surgery developed the disease afterwards (Fig. 5C,F). Among these, one maintained a BMI > 30 after bariatric and the other developed hyperglycemia at T2; the median time between surgeries was 27 months.

**Figure 6:**
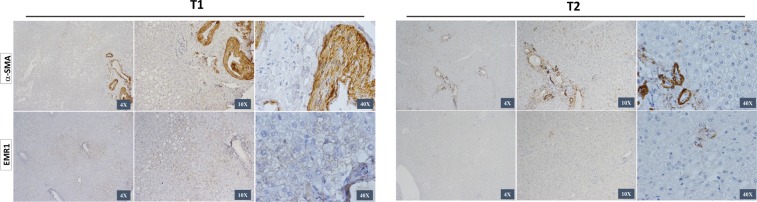
Immunostaining of liver biopsies at the time (T1) and after (T2) bariatric surgery showed Kupffer cell infiltration and myofibroblast formation. Representative images of the immunohistochemical analysis of EGF-like molecule containing mucin-like hormone receptor 1 (EMR1) and alpha-smooth muscle actin (α-SMA) were performed on paraffin-embedded slides from the T1 and T2 groups. Positive cells for immunostaining are shown in brown. 4×, 10× and 40× objective lenses were used as indicated in each image.

To better evaluate the hepatic lesions along the follow-up after the bariatric surgery in the paired cohort, we applied immunohistochemistry for alpha-smooth muscle actin (α-SMA) and for a specific cell-surface marker for macrophages (EMR1) highly and constitutively expressed on Kupffer’s cells in the liver, besides H&E and Masson’s Trichrome staining. The representative images of immunohistochemistry for Kupffer’s cell (EMR1) and myofibroblast formation (α-SMA) markers also illustrate NAFLD regression (Fig. 6).

## Discussion

The prevalence of overweight and obesity has steadily increased in the recent decades, regardless of gender, age or level of country development. For this reason, obesity has been considered a major public health problem worldwide. There are more than 2 billion overweight people and 64% of them are in developing countries^15^.

A successful public health model has not been yet identified to reverse this epidemic, mainly because it is a multifactorial disease, which depends on changes in lifestyle, such as less caloric intake and greater energy expenditure^16,17^. In the last decades, the relevance of the intestinal microbiota has been suggested as another possible factor involved in the increase of obesity. However, its impact on the treatment of obesity has been negligible^18^. Special attention should be given to children, due to the considerable reduction in life expectancy and quality related to obesity^19^. While overweight and obesity showed an increase in prevalence to 27.5% among adults, the infant population’s prevalence reached the high rate of 47.1% between 1980 and 2013^15^.

Brazil is the fifth nation in absolute number of obese individuals, behind the USA, China, India and Russia^15^. These data have justified the increased investment in public health for the prevention and treatment of obesity and its comorbidities. Obesity is related to several diseases, such as cardiovascular, orthopedic, diabetes and several types of cancer^20^, being associated with 14–20% of all cancer deaths^21^. Obesity is the main risk factor for NAFLD, while other factors such as ethnicity (prevalence of 45% in Hispanics; 33% in whites; 24% in blacks), gender (prevalence of 42% in white men; 24% in white women), type 2 diabetes (22.51%), hyperlipidemia (69.16%), hypertension (39.34%), and metabolic syndrome (42.54%) has less impact^22–24^. NAFLD is considered the hepatic phenotype of metabolic syndrome, which is directly related with insulin resistance and disturbances in glucose and lipid metabolism. The high worldwide incidence of NAFLD and its correlation with obesity and its comorbidities emphasize the importance to evaluate the impact of the hepatic features in this affection. To our knowledge, there is no other study that has identified and correlated such detailed aspects of the NAFLD in a large Brazilian cohort. All individuals who underwent bariatric surgery in this study participated in a preoperative weight loss program which lasts 4 to 12 weeks, includes weekly follow-ups, and is carried out by a multidisciplinary team. Individuals underwent surgery once a minimum of 10% preoperative weight loss was achieved, or with the minimal BMI of 35 kg/m^2^ for subjects with obesity-related morbidities or 40 kg/m^2^ for those free of comorbidities. This may justify the low mortality rate (0.33%) after bariatric surgery in this study, since the weight loss may assist to attenuate the associated comorbidities.

Bariatric surgery has largely evolved over time and nowadays its perioperative mortality is arguably as low as 0.2%, which is similar to what is observed after routinely performed operations such as gallbladder removal and hysterectomy, and leads to an overall 40% reduction of all-cause mortality^25,26^. Nonetheless, it is far from being free of risk, although the incidence of major complications reduced over time as well, mostly after the development of minimally invasive approaches. Early morbidity such as anastomotic leaks and thromboembolic events may occur in up to 2–4% of all operated individuals and, as such, are the most dreaded perioperative complications^27,28^. Late morbidity is more related to nutritional issues, such as protein, iron, and vitamin deficiencies, which are also associated with a poor compliance to follow-up, as well as surgical complications, such as intestinal obstruction caused by adhesions or internal hernias, abdominal wall hernias and gallstones. Significant hepatic impairment is unusual after the mainstream procedures, but there are a number of cases after more malabsorptive operations, mainly the biliopancreatric diversions^29–31^.

Described only in the 1980s, NALFD is currently considered the main liver disease worldwide, with prevalence in the general population of up to 25%, and analyzing only obese patients, the prevalence exceeds 50%^24,32–35^. The prevalence of liver diseases secondary to alcohol consumption remained stable, and the better diagnosis and subsequent development of vaccine and treatment for viral hepatitis decreased the liver injury due to this affection worldwide in the recent decades. On the other hand, liver disease associated with obesity and the consequent evolution to steatosis, steatohepatitis, cirrhosis and hepatocellular carcinoma has become one of the main causes of liver failure and transplantation in recent years, with the aggravating possibility of recurrence in the transplanted organ^36,37^. In our cross-sectional retrospective study, the NAFLD incidence evaluated by histological examination was 46.48% among the morbidly obese patients who underwent bariatric surgery, and 50% in the first timepoint of the paired longitudinal study. This incidence in bariatric patients was verified in other studies with smaller sample sizes^38–43^. While most of the studies evaluated NAFLD after bariatric surgery by ultrasound scan or another image or laboratory methods, our group performed liver biopsy at two distinct timepoints. Some serum levels of liver function markers were decreased in T2 compared to T1, that corroborate with the improvement of NAFLD verified in histological analysis in T2. Thus, there was a decrease in ALT (p = 0.0117), GGT (p = 0.0072), TGC (p = 0.0216) and COL (p = 0.0031) serum levels after bariatric surgery. The decreased serum albumin (p = 0.0014) and protein levels (p = 0.0109) may be explained by the decrease of protein intake and absorption after bariatric surgery.

Since steatosis (NAFL) and steatohepatitis (NASH) are asymptomatic in the vast majority of patients, we have a population of a few billion inhabitants with such diseases without a proper diagnosis^44^. This condition is aggravated because although the evolution of these changes towards liver cirrhosis and hepatocellular carcinoma is defined^24^, unfortunately it is not yet possible to predict which patients will remain stable and which will progress to cirrhosis and cancer. Since access to diagnostic tests is scarce in our country for a large part of the population, several patients receive their diagnosis only after the secondary symptoms of liver failure and cirrhosis appear.

Although bariatric surgery provides NAFLD amelioration, the literature is controversial about what surgical technique is more effective or may be equally efficacious in ameliorating NAFLD. All the patients included in this study underwent the same surgical procedure (RYGB bariatric surgery), and the weight loss outcomes were comparable to the literature^45^. Only 18% of the 895 patients included in the study were considered diabetic at the time of surgery, thus the histopathological changes found and corresponding liver damage may be attributed to the morbid obesity.

Given the importance of assessing liver function and morphology in susceptible patients, several methods have been developed, such as laboratory tests and indexes based on these tests, in addition to imaging tests such as ultrasound scan, nuclear magnetic resonance imaging and elastography^46^. As a screening method, ultrasound scan is a non-invasive, easily accessible and low-cost method to diagnose patients with hepatic steatosis, associated with the investigation of other causes of liver disease such as viral hepatitis, alcohol consumption and hepatotoxic drugs. However, liver biopsy, despite being invasive and not without complications, has been considered the gold standard for diagnosis of the disease and is the only method able to differentiate non-alcoholic steatohepatitis from steatosis, to evaluate inflammation and to stage fibrosis^46^.

In addition to the evaluation of NAFLD’s prevalence among morbidly obese patients who were submitted to the RYGB bariatric surgery in a large cohort, the present study identified the histological grading and evolution of the hepatic lesions in a subset of patients. There was a significant reduction of collagen fiber deposition in the liver biopsies after bariatric surgery, besides decreased hepatic steatosis and hepatocyte ballooning, showing NAFLD regression after the procedure. Moreover, immunohistochemistry for α-SMA and EMR1 corroborated these findings. α-SMA is expressed by hepatic stellate cells, reflects their activation to myofibroblast-like cell and has been directly related to experimental liver fibrogenesis and to human fibrosis in chronic liver disease^47^. In this study, we identified the earliest stages of hepatic fibrosis in morbid obese patients during and after bariatric surgery, showing positive staining to α-SMA in both timepoints, but less evident in T2 when compared to T1. Likewise, the staining for Kupffer cells marker (EMR1) showed positivity in both groups, but less in T2 than in T1. Kupffer cells are central players in the progression of steatosis to non-alcoholic steatohepatitis (NASH) and fibrosis^48^. Therefore, the immunohistochemistry analysis illustrated the results of the histopathological changes classification of the NAFLD based on H&E and Masson’s Trichome staining during and after the bariatric surgery. These findings better characterized the NAFLD histological regression that occurred in the majority of the cases of our longitudinal paired study.

Most of this study’s limitations are related to its retrospective character. Some variables for the applied histological score were not evaluated by the pathologists and, therefore, were not in the medical records, having been considered not informed, as pointed out in the Supplementary Information Files. Unfortunately, the late follow-up of bariatric surgery is reportedly erratic, since most patients do not appropriately comply with the appointed consultations^49^. This general limitation of the bariatric practice was also present within the current study, since the individuals which underwent a second biopsy were not regularly accompanied and thus there are no available data in many time points except for both perioperative situations. However, this cohort has a decent size, and a longitudinal dataset as the one presented is of value for the scientific community, as it shows results of liver histology after bariatric surgery. The preoperative weight loss may also have influenced the histological findings and the relatively low prevalence of NAFLD in the entire study population, as well as explain the low BMI at surgery in comparison with other studies.

The blood liver function tests may to estimate, preoperatively, patients who are prone to unfavorable evolution after bariatric surgery. However, the liver biopsy collected during the bariatric surgery with specific histological analysis may better identify the different types of lesions and map the patients with high risk for hepatic complications in the follow-up after this procedure. Moreover, this histologically focused study may bring new insights on the effects of weight loss in the NAFLD regression. There are a few cases of bariatric patients in the literature who develop hepatic injury after the procedure, and this observation needs further investigation based on the molecular and metabolic pathways involved in this process. Based on the currently available evidence, the occurrence of severe liver disease is unusual and even anecdotal after the mainstream bariatric procedures, but not so rare after predominantly malabsportive operations. The mechanisms of this worsening are yet to be determined, but seem to be related to early rapid weight loss, a degree of protein malnutrition, the lack of hepatotrophic factors, and the effect of high levels of mobilized circulating free fatty acids after surgery, as well as changes in gut microbiota and intestinal bacterial overgrowth^50–52^. The benefit of a routine second liver biopsy at a determined timepoint of the follow-up after bariatric surgery should be confirmed in future large longitudinal prospective studies; however, given the extremely low risk of liver biopsy complications in individuals who would otherwise have to undergo another surgery after their primary bariatric procedure (hernioplasty, cholecystectomy etc.), this seems to be justifiable within this context.

## Methods

### Patient and Sample Selection

We retrospectively analyzed the clinical and histopathological data from 954 Brazilian patients who underwent Roux-en-Y gastric bypass (RYGB) bariatric surgery in the Clinical Hospital of the University of Campinas between the years 2008 and 2019, by the same surgical team. Bariatric surgery was indicated according to the National Institutes of Health consensus statement. The patients included in this observational study had a BMI equal to or greater than 30 at the time of surgery. The wedge liver biopsy was performed at the time of bariatric surgery.

All bariatric procedures followed the same technique and were performed by an open approach. The main features of the Roux-en-Y gastric bypass (RYGB) were a 30 mL gastric pouch, a 100-cm biliopancreatic loop, a 150-cm alimentary limb and a common channel comprised of the remainder of the small intestine.

For the longitudinal observational prospective study, 30 patients who underwent bariatric surgery (T1) and a second abdominal surgical procedure after bariatric surgery (T2), such as cholecystectomy (7%), incisional hernia correction (50%), cholecystectomy + incisional hernia correction (3%), exploratory laparotomy (37%) and removal of gastric band (3%), were submitted to another liver biopsy during this procedure.

Clinical and demographic data, laboratory records and liver biopsy histopathological findings were collected from both cohorts. Histologic parameters to classify the NAFLD were evaluated to analyze the effect of bariatric surgery and weight loss on the evolution of this liver disease. NAFLD was estimated by hematoxylin and eosin (H&E) and Masson’s Trichrome staining, using the histopathological score recommended by the American Association for the Study of Liver Diseases (AASLD) and the European Association for the Study of the Liver (EASL)^13^ for both cohorts of patients. The specifications for the adopted criteria are detailed in the Supplementary Information File. Immunohistochemical staining of the liver biopsies from patients of the longitudinal cohort were performed to better characterize the NAFLD. These data correlate with liver disease stage and overtime evolution.

The exclusion criteria of the study were as follows: aged below 18 or over 80 years old; history of alcohol abuse; use of hepatotoxic drugs; history of chronic viral hepatitis and primary liver disease. In addition to these criteria, patients with BMI equal to or greater than 30 were excluded from the control group. All procedures performed in studies involving human participants were in accordance with the Declaration of Helsinki and its later amendments. The Ethics Committee from the University of Campinas approved the study (reference number:58184516.2.0000.5404). The study was carried out at the University of Campinas, Surgery Department and IBD Research Laboratory of the School of Medical Sciences.

### Tissue staining

Paraffin-embedded liver blocks of 9 patients who underwent bariatric surgery (T1) and a second abdominal surgery in their follow-up (T2) were used for staining assays. For histological analysis, 4 µm-thick sections were stained with hematoxylin and eosin (H&E) and Masson’s Trichrome dye. Photomicrographs were taken using Zeiss Axioplan 2 microscope with digital camera (Olympus DP – 72) and control software (Cellsens). Fields of higher magnification (4, 10 and 40×) were scanned and three random fields of higher magnification were selected for quantitative analysis, which was performed through the percentage of staining pixel volume in a panchromatic objective field of higher magnification 4× by ImageJ2 software.

### Immunohistochemical staining

Paraffin-embedded liver blocks of 12 patients who underwent bariatric surgery (T1) and a second abdominal surgery in the follow-up (T2) were used for immunostaining. For this, 4 µm-thick sections were pre-treated for de-paraffinization, rehydration, and epitope retrieval using citrate buffer. A warming step of 20 min at 95 °C was performed. Sections were blocked for 45 min with 1% bovine serum albumin (BSA) or with animal-free blocking solution (Vector Laboratories). Samples were incubated overnight at 4 °C using the following commercially available antibodies: anti-human EGF-like molecule containing mucin-like hormone receptor 1 (1:50; sc-52664;Santa Cruz Biotechnology) and anti-human Smooth Muscle Actin (1:100; M085101-2; Dako- Agilent Technologies) followed by incubation with biotinylated anti-mouse (1:200; Vector Laboratories) or anti-rat (1:200; Vector Laboratories). Signal detection was determined using the immunoperoxidase detection system (Vector Laboratories), followed by incubation with 3,3’-diaminobenzidine (DAB) solution (Dako). Slides were mounted with Mounting Medium (Dako). Photomicrographs were taken using Zeiss Axioplan 2 microscope with digital camera (Olympus DP – 72) and control software (Cellsens). Fields of higher magnification (4, 10 and 40×) were scanned.

### Statistical analysis

The results were reported as median with interquartile ranges. To test for distributional adequacy, the Kolmogorov-Smirnov test was used to investigate if the data followed a normal distribution or a Gaussian distribution (p > 0.1). Data were analyzed using the non-parametric Mann-Whitney test. The level of significance was set at p < 0.05.

### Consent for publication

Ethical approval by the ethic board of the University of Campinas (UNICAMP) and consent of patients are included in the original publications.

### Ethics approval and consent to participate

This study was approved by the Ethics Committee of the University of Campinas (UNICAMP), all patients signed the informed consent form, and were performed in accordance with the Declaration of Helsinki.

## Supplementary information

Supplementary information.

Supplementary information.

Supplementary information.

## References

[1] Huang, T.D., Behary, J. & Zekry, A. Non-alcoholic fatty liver disease (NAFLD): a review of epidemiology, risk factors, diagnosis and management. *Intern. Med. J*. 10.1111/imj.14709 (2019). 10.1111/imj.14709 31760676

[2] Ahmed IA, Mikail MA, Mustafa MR, Ibrahim M, Othman R (2019). Lifestyle interventions for non-alcoholic fatty liver disease. Saudi J. Biol. Sci..

[3] Reid AE (2001). Nonalcoholic steatohepatitis. Gastroenterology.

[4] Day CP, James OF (1998). Steatohepatitis: a tale of two “hits”?. Gastroenterology.

[5] Perumpail BJ (2017). Clinical epidemiology and disease burden of non-alcoholic fatty liver disease. World J. Gastroenterol..

[6] Bedogni G, Gastaldelli A, Foschi FG (2020). Fatty liver, cardiometabolic disease and mortality. Curr. Opin. Lipidol..

[7] Coccia F (2020). Insulin resistance, but not insulin response, during oral glucose tolerance test (OGTT) is associated to worse histological outcome in obese NAFLD. Nutr. Metab. Cardiovasc. Dis..

[8] Younossi ZM, Marchesini G, Pinto-Cortez H, Petta S (2019). Epidemiology of non-alcoholic fatty liver disease and non-alcoholic steatohepatitis: implications for liver transplantation. Transplantation..

[9] Sjostrom CD (2003). Surgery as an intervention for obesity. Results from the Swedish obese subjects study. Growth Horm. IGF Res..

[10] MacDonald KG (1997). The gastric bypass operation reduces the progression and mortality of non-insulin-dependent diabetes mellitus. J. Gastrointest. Surg.

[11] Albrecht RJ, Pories WJ (1999). Surgical intervention for the severely obese. Best Pract. Res. Clin. Endocrinol. Metab..

[12] Ratziu V (2000). Liver fibrosis in overweight patients. Gastroenterology.

[13] Rinella ME (2019). Report on the AASLD/EASL Joint Workshop on Clinical Trial Endpoints in NAFLD. Hepatology.

[14] Brown GT, Kleiner DE (2016). Histopathology of Non-alcoholic Fatty Liver Disease and Non-alcoholic Steatohepatitis. Metabolism..

[15] Ng M (2014). Global, regional, and national prevalence of overweight and obesity in children and adults during 1980-2013: a systematic analysis for the Global Burden of Disease Study 2013. Lancet.

[16] Follow-up to the Political Declaration of the High-level Meeting of the General Assembly on the Prevention and Control of Non-Communicable Diseases; Geneva, Switzerland: World Health Assembly., http://apps.who.int/gb/ebwha/pdf_files/WHA66/A66_R10-en.pdf (2013).

[17] Bleich S, Cutler D, Murray C, Adams A (2008). Why is the developed world obese? Ann. Rev. Public Health.

[18] Turnbaugh PJ (2009). A core gut microbiome in obese and lean twins. Nature..

[19] Ogden CL, Carroll MD, Kit BK, Flegal KM (2014). Prevalence of childhood and adult obesity in the United States, 2011-2012. JAMA..

[20] Lim SS (2012). A comparative risk assessment of burden of disease and injury attributable to 67 risk factors and risk factor clusters in 21 regions, 1990-2010: a systematic analysis for the Global Burden of Disease Study 2010. Lancet..

[21] Calle EE, Rodriguez C, Walker-Thurmond K, Thun MJ (2003). Overweight. Obesity, and Mortality from Cancer in a Prospectively Studied Cohort of U.S. Adults. N. Engl. J. Med..

[22] Vernon G, Baranova A, Younossi ZM (2011). Systematic review: the epidemiology and natural history of non-alcoholic fatty liver disease and non-alcoholic steatohepatitis in adults. Aliment. Pharmacol. Ther..

[23] Browning JD (2004). Prevalence of hepatic steatosis in an urban population in the United States: impact of ethnicity. Hepatology.

[24] Younossi ZM (2016). Global epidemiology of nonalcoholic fatty liver disease-Meta-analytic assessment of prevalence, incidence, and outcomes. Hepatology..

[25] Rausa E (2016). Rate of death and complications in laparoscopic and open Roux-en-Y Gastric Bypass. A Meta-analysis and Meta-regression Analysis on 69,494 Patients. Obes. Surg..

[26] Adams TD (2007). Long-term mortality after gastric bypass surgery. N. Engl. J. Med..

[27] Schulman AR, Thompson CC (2017). Complications of bariatric surgery: What you can expect to see in your GI practice. Am. J. Gastroenterol..

[28] Chang SH (2018). Early major complications after bariatric surgery in the USA, 2003-2014: a systematic review and meta-analysis. Obe.s Rev.

[29] Hamdan K, Somers S, Chand M (2011). Management of late postoperative complications of bariatric surgery. Br. J. Surg..

[30] le Roux CW, Heneghan HM (2018). Bariatric surgery for obesity. Med. Clin. North Am..

[31] Cazzo E, Pareja JC, Chaim EA (2017). Liver failure following biliopancreatic diversions: a narrative review. Sao Paulo Med. J..

[32] Jennings J, Faselis C, Yao MD (2018). NAFLD-NASH: An Under-Recognized Epidemic. Curr. Vasc. Pharmacol..

[33] Eslam M, Valenti L, Romeo S (2018). Genetics and epigenetics of NAFLD and NASH: Clinical impact. J. Hepatol..

[34] Tomic D, Kemp WW, Roberts SK (2018). Nonalcoholic fatty liver disease: current concepts, epidemiology and management strategies. Eur. J. Gastroenterol. Hepatol..

[35] Younossi Z (2018). Global burden of NAFLD and NASH: trends, predictions, risk factors and prevention. Nat. Rev. Gastroenterol. Hepatol..

[36] Bacon BR, Farahvash MJ, Janney CG, Neuschwander-Tetri BA (1994). Nonalcoholic steatohepatitis: an expanded clinical entity. Gastroenterology.

[37] Liu A (2018). Nonalcoholic Fatty Liver Disease: Epidemiology, Liver Transplantation Trends and Outcomes, and Risk of Recurrent Disease in the Graft. J. Clin. Transl. Hepatol.

[38] Silverman EM, Sapala JA, Appelman HD (1995). Regression of hepatic steatosis in morbidly obese persons after gastric bypass. Am. J. Clin. Pathol..

[39] Mattar, S.G., et al. Surgically-induced weight loss significantly improves nonalcoholic fatty liver disease and the metabolic syndrome. Ann. Surg. **242(4)**, 610-617 discussion 618–620 (2005). 10.1097/01.sla.0000179652.07502.3f PMC1402345 16192822

[40] Liu X, Lazenby AJ, Clements RH, Jhala N, Abrams GA (2007). Resolution of nonalcoholic steatohepatitis after gastric bypass surgery. Obes. Surg..

[41] Furuya CK, de Oliveira CP, de Mello ES, Sood GK (2007). Effects of bariatric surgery on nonalcoholic fatty liver disease: preliminary findings after 2 years. J. Gastroenterol. Hepatol.

[42] Moretto M, Kupski C, da Silva VD, Padoin AV, Mottin CC (2012). Effect of bariatric surgery on liver fibrosis. Obes. Surg..

[43] Vargas V (2012). Surgically induced weight loss by gastric bypass improves non-alcoholic fatty liver disease in morbid patients with obesity. World J. Hepatol..

[44] Karanjia RN (2016). Hepatic steatosis and fibrosis: Non-invasive assessment. World J. Gastroenterol..

[45] Wolfe BM, Kvach E, Eckel RH (2016). Treatment of Obesity: Weight Loss and Bariatric Surgery. Circ. Res..

[46] Sharma S, Khalili K, Nguyen GC (2014). Non-invasive diagnosis of advanced fibrosis and cirrhosis. World J. Gastroenterol..

[47] Carpino G (2005). Alpha-SMA expression in hepatic stellate cells and quantitative analysis of hepatic fibrosis in cirrhosis and in recurrent chronic hepatitis after liver transplantation. Dig. Liver Dis..

[48] Rosso C (2019). Crosstalk between adipose tissue insulin resistance and liver macrophages in non-alcoholic fatty liver disease. J. Hepatol..

[49] Hood MM (2016). Managing severe obesity: understanding and improving treatment adherence in bariatric surgery. J. Behav. Med..

[50] Keleidari B, Mahmoudieh M, Gorgi K, Sheikhbahaei E, Shahabi S (2019). Hepatic Failure After Bariatric Surgery: A Systematic Review. Hepat. Month.

[51] Addeo P, Cesaretti M, Anty R, Iannelli A (2019). Liver transplantation for bariatric surgery-related liver failure: a systematic review of a rare condition. Surg. Obes. Relat. Dis..

[52] Eilenberg M (2018). Significant Liver-Related Morbidity After Bariatric Surgery and Its Reversal-a Case Series. Obes. Surg..

