# Supplementary material for: Histological grading evaluation of non-alcoholic fatty liver disease after bariatric surgery: a retrospective and longitudinal observational cohort study
Source: Sci Rep. 2020 May 22;10:8496. doi: 10.1038/s41598-020-65556-2 (PMC7244764; doi:10.1038/s41598-020-65556-2)
Supplement: Supplementary file 1 — Supplementary information. [file 41598_2020_65556_MOESM1_ESM.pdf]

Histological grading evaluation of non-alcoholic fatty liver disease after bariatric surgery: a retrospective and longitudinal observational cohort study

Authors: Felipe David Mendonça Chaim, Lívia Bitencourt Pascoal, Fábio Henrique Mendonça Chaim, Bruna Biazon Palma, Tiago Andrade Damázio, Larissa Bastos Eloy, Rita Carvalho, Everton Cazzo, Martinho Antônio Gestic, Murillo Pimentel Utrini, Marciane Milanski, Elinton Adami Chaim, Raquel Franco Leal.

Supplementary Information – Evaluation of nonalcoholic fatty liver disease (NAFLD) in obese patients who underwent bariatric surgery (n=895). Histopathological findings of the patients included in the cross-sectional retrospective study according to the recommendation of the American Association for the Study of Liver Diseases (AASLD) and the European Association for the Study of the Liver (EASL), 2019*.

EVALUATION OF NAFLD - NUMBER OF PATIENTS ACCORDING TO HISTOPATHOLOGICAL CHANGES FOUND

(TOTAL NUMBER OF PATIENTS ASSESSED = 895)

Score	Steatosis	Ballooning	Lobular inflammation	Fibrosis
0	480	515	538	229
1	309	184	229	1A (n=295); 1B (n=18); 1C (n=37)
2	86	193	97	277
3	20	---	---	35
4	---	---	---	3
UNINFORMED	0	3	31	1

* Rinella M.E., et al. Report on the AASLD/EASL Joint Workshop on Clinical Trial Endpoints in NAFLD. Hepatology. 70(4):1424-1436 (2019).
