# Supplementary material for: Histological grading evaluation of non-alcoholic fatty liver disease after bariatric surgery: a retrospective and longitudinal observational cohort study
Source: Sci Rep. 2020 May 22;10:8496. doi: 10.1038/s41598-020-65556-2 (PMC7244764; doi:10.1038/s41598-020-65556-2)
Supplement: Supplementary file 2 — Supplementary information. [file 41598_2020_65556_MOESM2_ESM.pdf]

**Histological grading evaluation of non-alcoholic fatty liver disease after bariatric surgery: a retrospective and longitudinal observational cohort study**

**Authors:** Felipe David Mendonça Chaim, Lívia Bitencourt Pascoal, Fábio Henrique Mendonça Chaim, Bruna Biazon Palma, Tiago Andrade Damázio, João José Fagundes, Larissa Bastos Eloy, Everton Cazzo, Martinho Antônio Gestic, Murillo Pimentel Utrini, Marciane Milanski, Raquel Franco Leal, Elinton Adami Chaim.

**Supplementary Information – Evaluation of nonalcoholic fatty liver disease (NAFLD) in obese patients who underwent bariatric surgery (T1) and a second abdominal surgery in the follow-up (T2) (n=30).** Histopathological findings of the patients included in the longitudinal study according to the recommendation of the American Association for the Study of Liver Diseases (AASLD) and the European Association for the Study of the Liver (EASL), 2019*.

**EVALUATION OF NAFLD - NUMBER OF PATIENTS ACCORDING TO HISTOPATHOLOGICAL CHANGES FOUND**

| | Score | Steatosis | Ballooning | Lobular inflammation | Fibrosis |
|---|---|---|---|---|---|
| Prebariatric Patients (T1) | 0 | 15 | 15 | 17 | 5 |
| | 1 | 10 | 8 | 9 | 1A (n=8); 1B (n=2); 1C (n=5) |
| | 2 | 5 | 7 | 4 | 9 |
| | 3 | 0 | 0 | 0 | 1 |
| | 4 | --- | --- | --- | 0 |
| Postbariatric patients (T2) | 0 | 25 | 22 | 23 | 7 |
| | 1 | 5 | 4 | 4 | 1A (n=10); 1B (n=3); 1C (n=1) |
| | 2 | 0 | 4 | 3 | 7 |
| | 3 | 0 | 0 | 0 | 2 |
| | 4 | --- | --- | --- | 0 |

* Rinella M.E., et al. Report on the AASLD/EASL Joint Workshop on Clinical Trial Endpoints in NAFLD. Hepatology. 70(4):1424-1436 (2019).
