# Supplementary material for: Histological grading evaluation of non-alcoholic fatty liver disease after bariatric surgery: a retrospective and longitudinal observational cohort study
Source: Sci Rep. 2020 May 22;10:8496. doi: 10.1038/s41598-020-65556-2 (PMC7244764; doi:10.1038/s41598-020-65556-2)
Supplement: Supplementary file 3 — Supplementary information. [file 41598_2020_65556_MOESM3_ESM.pdf]

Histological grading evaluation of non-alcoholic fatty liver disease after bariatric surgery: a retrospective and longitudinal observational cohort study

Authors: Felipe David Mendonça Chaim, Lívia Bitencourt Pascoal, Fábio Henrique Mendonça Chaim, Bruna Biazon Palma, Tiago Andrade Damázio, Larissa Bastos Eloy, Rita Carvalho, Everton Cazzo, Martinho Antônio Gestic, Murillo Pimentel Utrini, Marciane Milanski, Elinton Adami Chaim, Raquel Franco Leal.

Supplementary Information - Score to evaluate nonalcoholic fatty liver disease (NAFLD). A. Adopted criteria to classify histopathological findings according to the recommendation of the American Association for the Study of Liver Diseases (AASLD) and the European Association for the Study of the Liver (EASL), 2019*.

FLIP (Fatty Liver Inhibition of Progression) Steatosis, Activity, and Fibrosis Score

Histological Feature	Score	Category	Definition
Steatosis	0-3	0	<5%
		1	5% - 33%
		2	34% - 66%
		3	>66%
Ballooning	0-2	0	None
		1	Hepatocytes with rounded shape and pale cytoplasm
		2	Same as grade 1 with enlarged hepatocytes (>2x normal size)
Lobular inflammation	0-2	0	None
		1	<2 foci per ×20 field
		2	>2 foci per ×20 field
Fibrosis		0	No fibrosis
		1a	Zone 3 mild perisinusoidal
		1b	Zone 3 moderate perisinusoidal
		1c	Periportal/portal
		2	Zone 3 plus portal/periportal
		3	Bridging
		4	Cirrhosis

* Rinella M.E., et al. Report on the AASLD/EASL Joint Workshop on Clinical Trial Endpoints in NAFLD. Hepatology. 70(4):1424-1436 (2019).
